# LITTLE MEMORY EDITORS LIVING INSIDE YOUR BRAIN

**DOI:** 10.3389/frym.2023.968856

**Published:** 2023-02-22

**Authors:** Jie Zheng, Gordon Chen, Gabriel Kreiman, Ueli Rutishauser

**Affiliations:** 1 Department of Ophthalmology, Harvard Medical School, Boston Children’s Hospital, Boston, MA, United States; 2 Department of Biochemistry and Molecular Biology, Boston University, Boston, MA, United States; 3 Center for Brains, Minds and Machines, Cambridge, MA, United States; 4 Department of Neurosurgery, Cedars-Sinai Medical Center, Los Angeles, CA, United States; 5 Department of Neurology, Cedars-Sinai Medical Center, Los Angeles, CA, United States; 6 Department of Biomedical Sciences, Center for Neural Science and Medicine, Cedars-Sinai Medical Center, Los Angeles, CA, United States; 7 Division of Biology and Biological Engineering, California Institute of Technology, Pasadena, CA, United States

## Abstract

We interact with the world continuously. However, memories of our experiences are stored as individual events. For example, when we go on a road trip, we do not remember what happens second by second. Instead, we remember only a few special moments or events from a trip, such as dancing around the campfire. Our brains constantly extract memorable events while we interact with the world, and we organize those events based on their relevance. This process is like grouping road trip photos under different folders on the computer, so we can efficiently and accurately retrieve those memories in the future. How does the brain create these memorable events? In this article, you will learn about two groups of neurons inside the brain that help achieve this remarkable feat. You will also learn about how the activation of these neurons shapes the formation and retrieval of memories.

## THE BRAIN SPLITS OUR EXPERIENCES INTO SEPARATE EVENTS

Imagine a typical school day. You attend classes in math, science, and language. Can you remember every sentence each teacher said? Probably not. But you will almost certainly remember something from each class. Maybe a mathematical equation, a puzzling scientific observation, a new grammar rule, or even a joke that a classmate told you in the middle of class. When you get home and your mom or dad asks about your day at school, how will you describe it? Will you tell them all the details of your day in the specific order that you experienced them? Probably not. In fact, you might have a hard time remembering whether the new joke happened before science class or after. This is because we extract only the important moments or events from what we experience and store them in memory [1].

It is harder to retrieve memory events across different situations or **contexts**, such as things that happened in math class vs. things that occurred in science class. This is related to the well-known “doorway” effect [2], in which a person’s memory of what happened in one room declines when they pass through a doorway into another room. This transition from one situation or context to another is called a **context shift**. Have you ever gone into a room with some purpose in mind, but forget what that reason was once you got there? If so, you experienced the “doorway” effect. So, context shifts mark the beginning and the end of an event, or in other words, the **event boundaries**. How do our brains detect event boundaries?

## NEURONS IN THE BRAIN MARK EVENT BOUNDARIES

**Figure 1:**
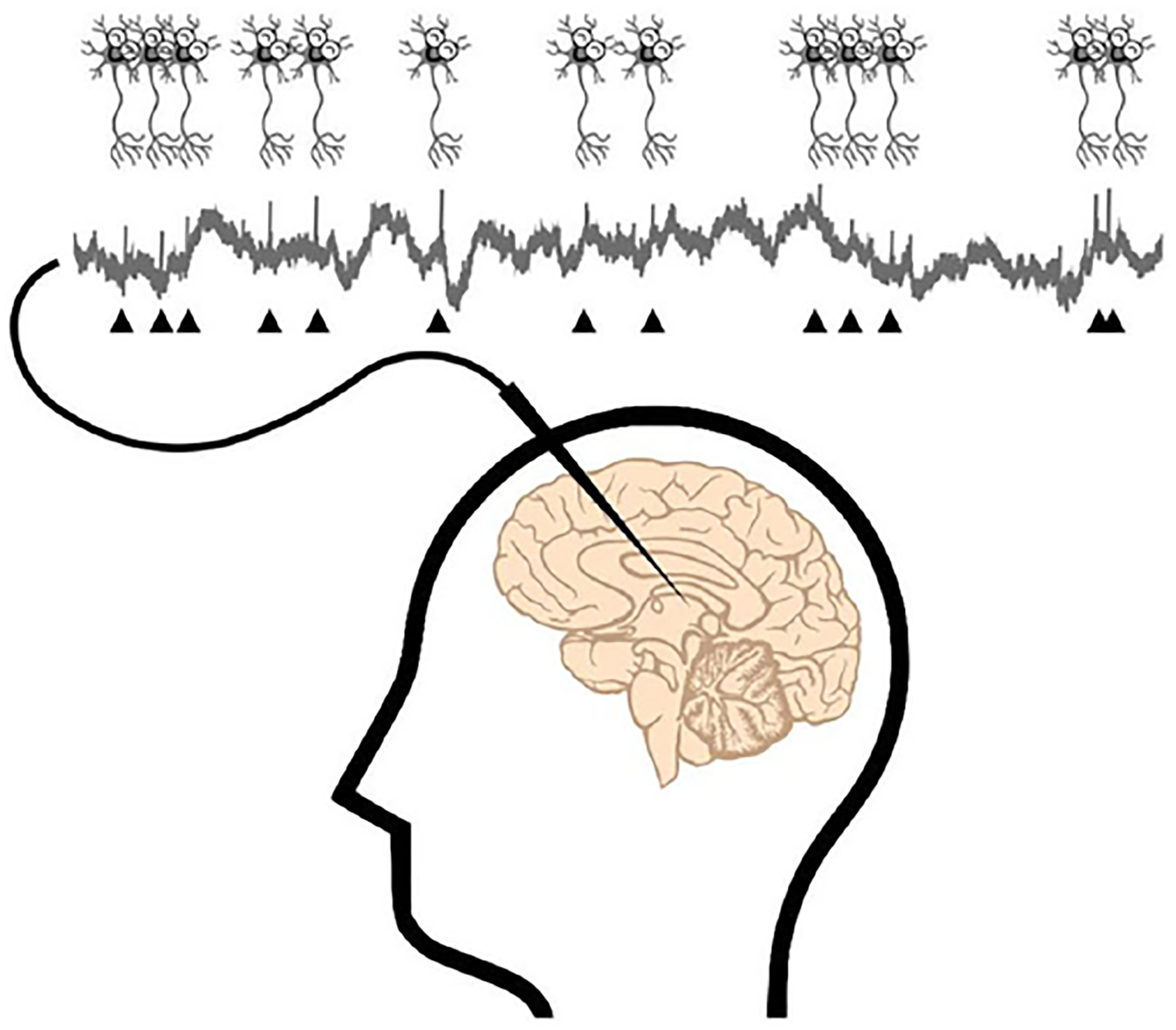
If electrodes (needle-shaped device) are implanted in the brain, we can measure the signals of the neurons next to the electrodes. The triangles mark the “uptick” in the measured signal due to neuron activation.

The brain is made up of cells called **neurons** that transmit and process information from the outside world (For more information on how the brain communicates with the body, see this Frontiers for Young Minds article). Think of neurons as existing in one of two states: activate or inactive. Neurons can switch between these two states at any moment. Twenty patients with a brain disorder called drug-resistant epilepsy volunteered to participate in our study (For more information on epilepsy, see this Frontiers for Young Minds article). These patients had measuring devices called **electrodes** implanted inside various brain regions, to diagnose which parts of the brain their seizures were coming from. For our study, we could also use these electrodes to “listen in” on the brain to understand how our brains detect event boundaries. When neurons near the electrode are active, we see small upticks (triangles in Figure 1) in the signals we record from the brain’s neurons.

**Figure 2:**
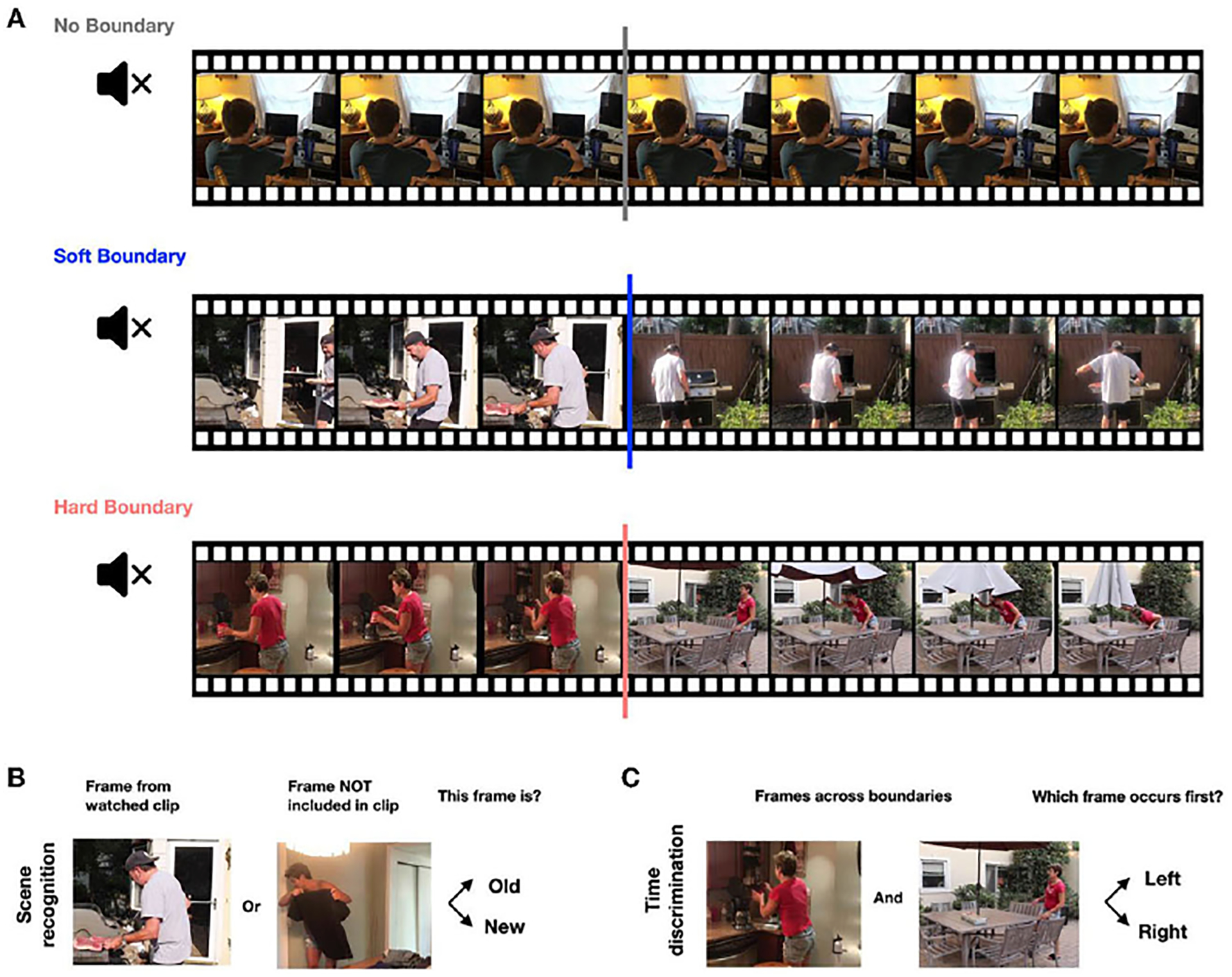
**(A)** Participants in our memory experiment watched a series of silent video clips with either no boundaries, soft boundaries, or hard boundaries. There were 90 total clips, 30 for each category. **(B)** After a 5-min break, participants performed a scene-recognition task. They were presented with single frames and asked to decide whether each was “old” (seen from watched clips) or “new” (not seen in watched clips). **(C)** Finally, participants performed a time-discrimination task. They were presented with pairs of frames and asked which frame occurred first in the watched clips.

While we monitored their neural signals, the participants did a memory experiment (Figure 2). They watched ninety silent video clips containing different kinds of boundaries. Clips with *no boundaries* contained continuous footage, without any editing. Clips with soft and hard boundaries both contained scene transitions, like you have probably seen between scenes in the movies. *Soft boundaries* cut to a related scene, but *hard boundaries* cut to a completely unrelated scene. For example, a scene of a person barbecuing that switches from a side view to a back view is a soft boundary, while a scene that changes from making coffee to folding an umbrella is a hard boundary (Figure 2A).

**Figure 3:**
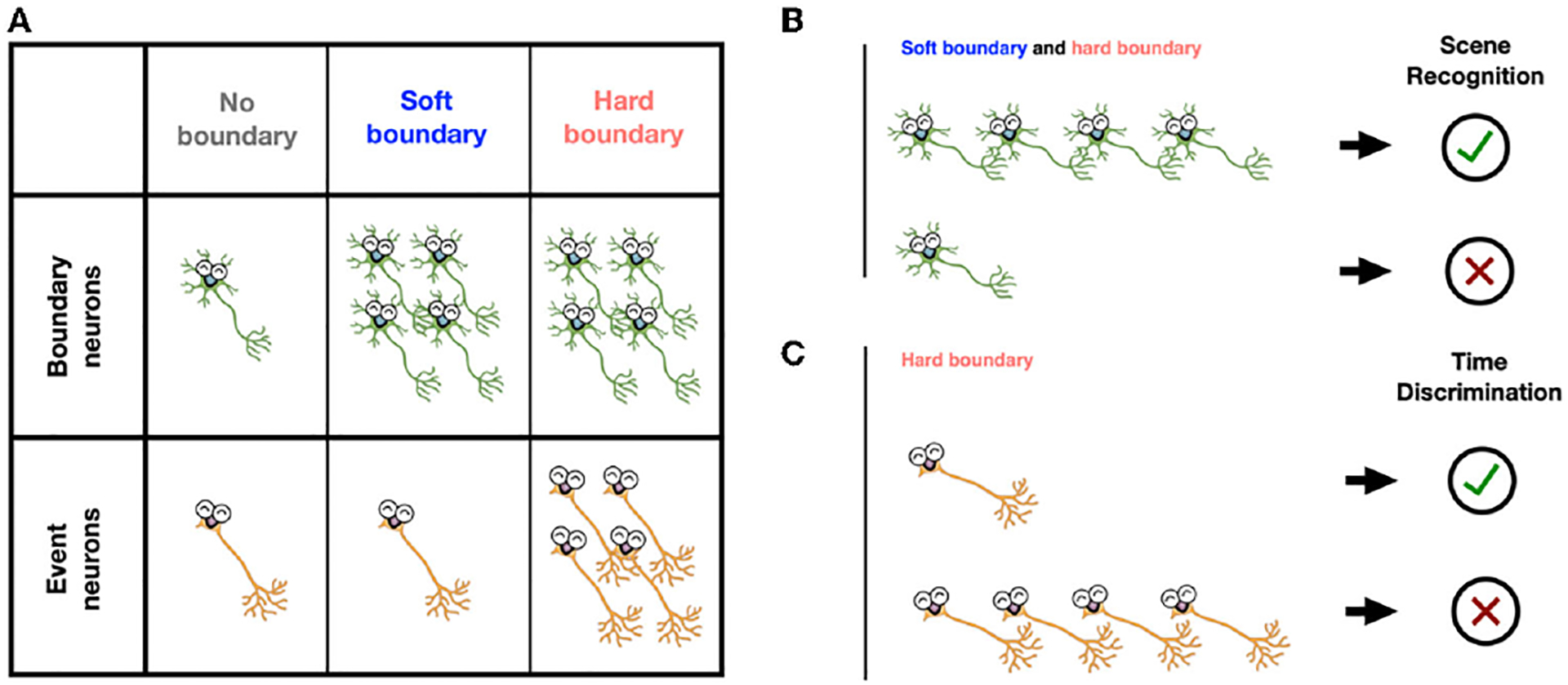
**(A)** During clip watching, boundary neurons activated after both soft boundaries and hard boundaries. Event neurons activated only after hard boundaries (more neurons in the figure indicate more activity). **(B)** *More* activation of boundary neurons after event boundaries led to better scene-recognition memory. **(C)** *Less* activation of event neurons at hard boundaries led to better time-order memory.

Is the brain aware of these different boundaries when watching a clip? Definitely! We found that two groups of neurons helped identify different kinds of event boundaries (Figure 3A). **Boundary neurons** activated a lot when participants watched video clips of both soft and hard boundaries, while **event neurons** only activated a lot at hard boundaries. Both types of neurons are primarily located in the **hippocampus**, a brain area responsible for making new memories.

## NEURON RESPONSES TO EVENT BOUNDARIES PREDICT MEMORY

How do neuron responses to event boundaries shape memory? To address this question, let us look at the second part of the memory game (Figures 2B,C). After watching all ninety clips, we tested participants’ memories about each clip in two different ways. The scene-recognition task helped assess how well participants recognized clip contents. Participants were presented with a single frame taken from one of the clips. They were asked to press the button and decide whether they had seen this frame (“old”) or not (“new”). The second test was a time-discrimination task, with which we tested whether participants could recall the correct order of the frames taken from videos they saw. Participants were presented with two frames side-by-side on the screen, one from before and one from after an event boundary. They were asked to choose which frame was seen earlier in the original clip.

For the scene-recognition task, we found that participants were better at recognizing the frames presented shortly after an event boundary. In contrast, frames that were further away from event boundaries were remembered less well. This suggests that the brain takes mental “snapshots” shortly after encountering an event boundary. In the time-discrimination task, it was more difficult for participants to decide which frame happened first when those frames were separated by a hard boundary. This is similar to the “doorway” effect discussed above.

Tying our findings together, we found that neural activation at event boundaries predicted how good the participants’ memories of the clips were. For a given clip, if boundary neurons activated a lot at soft and hard boundaries, participants were more likely to remember whether they had seen a frame from the clip or not (Figure 3B). Meanwhile, if event neurons activated less at hard boundaries, participants were more likely to remember the order of frames from their watched clips (Figure 3C). Hard boundaries helped establish memories of the event itself but made it hard to remember the order in which the events happened.

## WHY IS THIS IMPORTANT?

In this study, we used video clips to mimic real-life experiences and to study how human brains form and retrieve memories. We found that event boundaries shape our memories. Recognition memory is enhanced for things that happen right after event boundaries, while memory of the order of events is decreased by hard boundaries. We identified two groups of neurons that mark event boundaries. Boundary neurons activate at both soft and hard boundaries, while event neurons only respond to hard boundaries.

These findings demonstrate how brain neurons help break continuous experiences down into individual events—a fundamental but mysterious aspect of human memory. The effects that we observed have potential applications in the future. For example, boosting the activation of boundary neurons and event neurons might help people with memory disorders who have difficulty detecting event boundaries or remembering the order of events. Our findings also open a lot of interesting questions. Can boundary neurons and event neurons mark the event boundaries from an audio signal such as a podcast, for example? What happens in the brain to activate boundary neurons and event neurons? Why does the brain care about event boundaries? We hope that young scientists like you will help us answer all these questions in the future.

## Abbreviations

CONTEXT: The setting of an event, including what happens, where it happens, and when it happens.
CONTEXT SHIFT: Changes of event setting, including switching to a different subject, location, and topic.
EVENT BOUNDARY: The moment when switching from one event to another.
NEURON: Brain cells that receive sensory information from the external world and communicate with other neurons and the body.
ELECTRODE: A device that can record electrical activity from neurons.
BOUNDARY NEURONS: Neurons activated a lot at the soft and hard boundaries of video clips.
EVENT NEURONS: Neurons activated a lot at the hard boundaries of video clips.
HIPPOCAMPUS: A seahorse-shaped region deep inside the brain that is critical for memory. The name comes from the Greek: “hippos” meaning “horse,” and “kampos” meaning “sea monster.”
